# Atrial Functional Tricuspid Regurgitation as a Distinct Pathophysiological and Clinical Entity: No Idiopathic Tricuspid Regurgitation Anymore

**DOI:** 10.3390/jcm11020382

**Published:** 2022-01-13

**Authors:** Diana R. Florescu, Denisa Muraru, Valentina Volpato, Mara Gavazzoni, Sergio Caravita, Michele Tomaselli, Pellegrino Ciampi, Cristina Florescu, Tudor A. Bălșeanu, Gianfranco Parati, Luigi P. Badano

**Affiliations:** 1 Faculty of Medicine, University of Medicine and Pharmacy of Craiova, 200349 Craiova, Romania; dianarflorescu@yahoo.com (D.R.F.); cristina.t.florescu@umfcv.ro (C.F.); adrian.balseanu@umfcv.ro (T.A.B.); 2 Department of Cardiology, Istituto Auxologico Italiano, IRCCS, 20145 Milan, Italy; d.muraru@auxologico.it (D.M.); valevolpato@hotmail.it (V.V.); gavazzonimara@gmail.com (M.G.); s.caravita@auxologico.it (S.C.); m.tomaselli5@campus.unimib.it (M.T.); pellegrino.ciampi.rc@gmail.com (P.C.); gianfranco.parati@unimib.it (G.P.); 3 Department of Medicine and Surgery, University of Milano-Bicocca, 20126 Milan, Italy; 4 Department of Management, Information and Production Engineering, University of Bergamo, 24044 Dalmine, Italy; 5 Department of Cardiovascular and Pneumological Sciences, Catholic University of the Sacred Heart, 20123 Rome, Italy

**Keywords:** tricuspid regurgitation, atrial functional tricuspid regurgitation, transcatheter tricuspid valve interventions, echocardiography, three-dimensional echocardiography, multimodality imaging

## Abstract

Functional tricuspid regurgitation (FTR) is a strong and independent predictor of patient morbidity and mortality if left untreated. The development of transcatheter procedures to either repair or replace the tricuspid valve (TV) has fueled the interest in the pathophysiology, severity assessment, and clinical consequences of FTR. FTR has been considered to be secondary to tricuspid annulus (TA) dilation and leaflet tethering, associated to right ventricular (RV) dilation and/or dysfunction (the “classical”, ventricular form of FTR, V-FTR) for a long time. Atrial FTR (A-FTR) has recently emerged as a distinct pathophysiological entity. A-FTR typically occurs in patients with persistent/permanent atrial fibrillation, in whom an imbalance between the TA and leaflet areas results in leaflets malcoaptation, associated with the dilation and loss of the sphincter-like function of the TA, due to right atrium enlargement and dysfunction. According to its distinct pathophysiology, A-FTR poses different needs of clinical management, and the various interventional treatment options will likely have different outcomes than in V-FTR patients. This review aims to provide an insight into the anatomy of the TV, and the distinct pathophysiology of A-FTR, which are key concepts to understanding the objectives of therapy, the choice of transcatheter TV interventions, and to properly use pre-, intra-, and post-procedural imaging.

## 1. Introduction

Functional tricuspid regurgitation (FTR), secondary to tricuspid annulus (TA) dilation, tricuspid valve (TV) leaflet tethering, or a combination of both, resulting in leaflet malcoaptation [1], accounts for ~90% of all cases of TR [2,3]. FTR represents a progressive valvular condition that plays a strong and independent role in patient morbidity and mortality [4,5,6,7,8]. Several studies have demonstrated that, if left untreated, FTR can independently worsen patient outcomes and quality of life [4,9,10]. Furthermore, the development of transcatheter procedures to either repair or replace the TV [11], as valuable treatment alternatives in patients considered at high surgical risk, has further contributed to the increased interest in the pathophysiology, severity assessment, and clinical consequences of FTR [12,13,14,15,16,17].

FTR has been traditionally considered secondary to the dilation and/or dysfunction of the right ventricle (RV), mainly associated to pulmonary hypertension. Only recently, atrial FTR (A-FTR) has been recognized as a distinct pathophysiological entity, and its peculiar mechanisms have begun to being elucidated [18,19,20,21,22,23]. A-FTR is typically characterized by the dilation, and either the decrease or loss of the sphincter function of the TA, associated with the dilation and the dysfunction of the right atrium (RA), in patients with persistent/permanent atrial fibrillation (AF) [21,24,25]. These geometrical and functional changes determine an imbalance between the TA and leaflet areas, resulting in malcoaptation of the TV leaflets, even in the presence of normal RV size and function (type I of the Carpentier classification [26,27]). Given the distinct pathophysiological cascade leading to significant FTR development, A-FTR might pose different needs of clinical management [28], and the various interventional treatment options will likely have different outcomes than in the classical ventricular form of FTR (V-FTR, type IIIb of the Carpentier classification) [29,30]. Moreover, the transcatheter tricuspid valve interventions (TTVI) in A-FTR mainly have the goal to decrease the size of the TA, and require specific criteria of anatomic feasibility to plan the procedure [31,32,33].

Accordingly, the aim of this review is to provide an insight into the anatomy of the TV, and the pathophysiology of A-FTR, which are key concepts to understanding the objectives of therapy, the choice of TTVI, and to properly use pre-, intra-, and post-procedural imaging [32].

## 2. Anatomy and Pathophysiology of A-FTR

The TV is a complex structure that includes the TA, the TV leaflets, and a sub-valvular apparatus (chordae and papillary muscles). Both the anatomic integrity of the TV apparatus and the normal shape and function of the right heart chambers are needed for the correct functioning of the valve [31,34,35].

**Figure 1:**
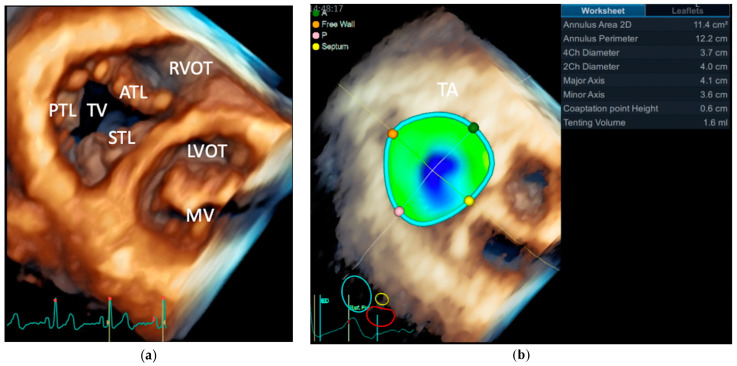
Transthoracic 3DE volume rendering of the tricuspid valve. (**a**) Anatomy of the tricuspid valve seen from the ventricular perspective and its relationships with adjacent structures; (**b**) Quantitative assessment of the tricuspid annulus. The colored dots on the annulus are used for anatomic orientation. Abbreviations: 2Ch, 2-chamber view; 2D, two-dimensional echocardiography; 3DE, three-dimensional echocardiography; 4Ch, 4-chamber view; A, anterior; ATL, anterior tricuspid leaflet; LVOT, left ventricular outflow tract; MV, mitral valve; P, posterior; PTL, posterior tricuspid leaflet; RVOT, right ventricular outflow tract; STL, septal tricuspid leaflet; TA, tricuspid annulus; TV, tricuspid valve.

The healthy TA has a dynamic, three-dimensional (3D) saddle-shaped elliptical geometry (Figure 1) [34,35,36,37], characterized by higher antero-septal and postero-lateral parts and lower antero-lateral and postero-septal parts [37].

The size of the TA is larger during diastole and smaller during systole. In pathological conditions of TA dilation, it tends to become more planar and circular [34]. The anterior and posterior parts of the TA are muscular, whereas the septal part is more fibrous. Consequently, the portion of the TA that is the least involved in the remodeling process is the septal one, the dilation mostly occurring in the antero-posterior direction, and leading to the progressive distancing of the aortic valve and the antero-posterior commissure [34].

However, the prevalence of significant FTR is extremely variable with the same degree of RA and TA dilation, and TA dilation secondary to RA remodeling in patients with persistent/permanent AF is not always associated with the development of significant FTR [38]. The imbalance between the degree of TA enlargement and the severity of A-FTR in some patients might partly be explained by the molecular adaptive mechanisms of the leaflet tissue that impact the amount of leaflet growth in response to the remodeling of the RA and the dilation of the TA, similar to those described in V-FTR [39,40]. Afilalo et al. [39] showed that in V-FTR, the remodeling and pressure overload of the RV are associated with a significant increase of the TV leaflet areas. The difference between the extent of TV leaflet areas adaptation in response to TA and RA dilation could be a key factor in the pathophysiological cascade that leads to the development and progression of A-FTR. Moreover, Utsunomiya et al. [19] showed that the posterior dilation of the RA that causes posterior TV plane displacement is not efficiently compensated by the TV leaflet adaptation. These mechanisms might explain why despite similar extent of RA dilation, some of the patients present with only trivial/mild A-FTR [21].

Although several studies have elegantly described the pathophysiological mechanisms of A-FTR (previously-referred to as “idiopathic” or “isolated” TR), it represents nevertheless an overlooked consequence of persistent/permanent AF. A-FTR is characterized by TA remodeling associated with RA enlargement, and normal/mildly abnormal RV size and function, especially in the initial stages of the disease [18]. Yamasaki et al. [41] hypothesized that severe A-FTR is caused by the loss of TV leaflets’ systolic coaptation in the context of TA and RA dilation. Muraru et al. [24,42] demonstrated that the RA plays a substantial role in determining TA size in FTR patients, including A-FTR. Guta et al. [21] have further contributed to understanding the pathophysiology of A-FTR by showing that RA minimum volume is the main determinant of TA area at end-diastole in AF patients, and that it determines A-FTR severity, while leaflet tethering plays a far less important role in the process. Furthermore, Utsunomiya et al. [43] showed that TA area was more closely correlated with RA maximum volume than with RV end-systolic volume in AF patients, and that the only predictor of A-FTR severity was TA area at mid-systole. In contrast, both RA and RV volumes were found to be independent predictors of severe A-FTR according to Najib et al. [44]. RV enlargement is usually detected in more advanced stages of A-FTR, with longer disease progression, as the dilation of the RV is usually a late event in A-FTR, as reported by Nemoto et al. [45]. Finally, the shape of the RV is markedly different in patients with A-FTR and V-FTR [23]. In A-FTR the RV remodeling pattern resembles a conical shape, with isolated enlargement of the inflow portion of the RV, and without significant chamber dilation or dysfunction compared to controls. Conversely, in V-FTR the RV becomes spherical or elliptic, with significantly increased basal and mid-cavity RV diameters and volumes, and significantly decreased RV function compared to controls [23]. Therefore, RV size assessment by two-dimensional echocardiography (2DE) linear methods, such as RV basal diameter, has important limitations in patients with A-FTR, and should be replaced by three-dimensional echocardiography (3DE) volumetric measurement.

## 3. TTVIs in A-FTR

Although still under development and underused in clinical practice, TV interventions should be considered in patients with severe symptomatic FTR, in the absence of severe left ventricular or RV dysfunction, or severe pulmonary hypertension (class IIa) [17,29], and according to current guidelines [16,46], RV dilation is a criterion for severe FTR. However, it has recently been reported that patients with severe A-FTR might present with normal RV size, and a dilated RV might be found in patients with less than severe A-FTR [23]. Therefore, A-FTR severity grading should be carefully performed, and absence of RV enlargement should not be considered an exclusion criterion of severe A-FTR. Moreover, FTR severity is not linearly associated with prognosis [47], demonstrating that the recommended indications for TV interventions should take into consideration the etiology of FTR [16]. In a recent study, patients with severe FTR treated with TTVIs had better 1 year prognosis compared to patients undergoing only medical treatment [48]. In patients with indications for TV interventions, diuretic therapy is useful in the presence of right-sided heart failure, and rhythm control strategies might decrease A-FTR severity in patients with AF [24,43,49,50,51]. Wang et al. [50] demonstrated that catheter ablation (CA) for AF and sinus rhythm (SR) maintenance lead to TR improvement in FTR patients without significant TV tethering (tethering height < 6 mm). These findings are supported by the study by Markman et al. [51] that show a significant reduction (of at least one grade) of TR severity in 64% of patients after CA for AF. Lastly, Itakura et al. [52] showed how the reduction in RA size following the restoration of SR by CA correlated with the decrease in FTR severity in patients with persistent AF. However, although cardioversion and/or ablation of AF might be beneficial in patients with A-FTR, these therapies should not delay the referral for intervention in patients with indications [16].

In patients referred for TTVIs, the parameters used for TR grading often have far greater values than the lower thresholds currently recommended to identify severe TR [53,54], and among all patients with functional atrioventricular valve regurgitation of various causes, patients with A-FTR can particularly have extremely severe annular dilation, making catheter-guided interventions challenging and controversial in end-stage forms [55]. These findings have highlighted the need for a novel grading system that could illustrate the continuum of TR severity [56]. A group of experts proposed the introduction of two new TR categories, massive and torrential TR, by extending the current cut-off values for severe TR [13,57], and their significance has been demonstrated in several studies [13,15,54,58]. The systematic combined use of vena contracta (VC) width and effective regurgitant orifice area (EROA) to identify severe (VC width ≥ 7 mm and EROA < 80 mm^2^) and torrential TR (VC width ≥ 7 mm and EROA ≥ 80 mm^2^) has been useful in predicting patient outcomes in significant FTR [59]. Since massive to torrential A-FTR is characterized by prominent annular dilation associated with significant tethering of the leaflets, Utsunomiya et al. [19] suggested that the most suitable patients for TR annuloplasty are those with severe FTR. Therefore, the updated proposed FTR severity grading could impact the timing of TV interventions, especially since they are mostly performed too late, in end-stage forms. Subsequently, TTVIs may improve the prognosis of patients with severe A-FTR, especially as an early treatment, before the development of massive to torrential FTR.

The feasibility, safety, and efficacy of TTVIs have been demonstrated in recent studies [53,60,61]. The best technique and choice of intervention are based on an accurate pre-procedural assessment consisting of multimodality imaging evaluation [31,62,63], yet relying mainly on echocardiography, and the identification of the exact mechanism of TR. To confirm the indication of TTVI and to select the type and size of the device used, accurate measurement of the TA done using 3D imaging (echocardiography, multidetector cardiac computed tomography- CCT, or cardiac magnetic resonance- CMR) is key [64]. Furthermore, a deep understanding of the anatomic relationships between the TV and various essential surrounding structures such as the right coronary artery, the conduction tissue, the aortic valve, and the coronary sinus (CS) are of paramount importance in planning, guiding, and monitoring of TTVIs [31,65,66].

### 3.1. Echocardiography

The state-of-the-art echocardiographic evaluation of the TV and quantification of the severity of FTR should imply: (1) confirming the presence of pathological FTR; (2) assessing the morphology of the TV; (3) identifying the mechanisms of FTR (annular dilation, leaflet tethering, cardiac implanted electronic device interference, etc.); (4) distinguishing between A-FTR and V-FTR; (5) assessing the severity of FTR and quantifying its hemodynamic impact [67].

**Figure 2:**
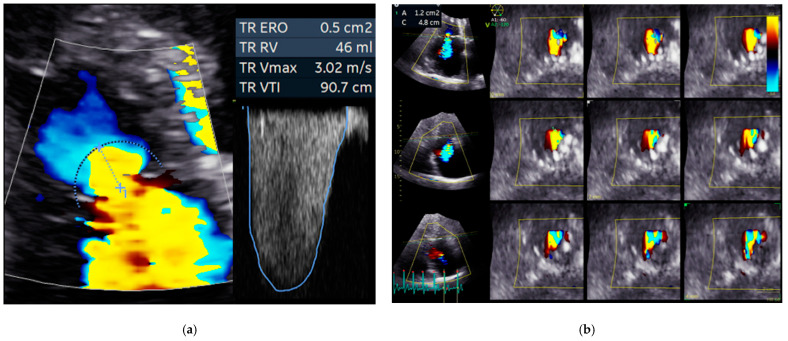
Quantitative assessment of functional tricuspid regurgitation severity by Color-Doppler echocardiography. (**a**) 2D PISA method. (**b**) 3D vena contracta area. Abbreviations: 2D, two-dimensional echocardiography; 3D, three-dimensional echocardiography; A, area; C, circumference; ERO, effective regurgitant orifice area; RV, right ventricle; TR, tricuspid regurgitation; Vmax, maximal regurgitant velocity; VTI, velocity-time integral.

In clinical routine practice, 2DE and Doppler echocardiography are recommended by guidelines for TR evaluation [26,68,69]. When quantifying TR severity, different parameters (structural, qualitative, semi-quantitative, or quantitative) should be evaluated (Figure 2), and grading of FTR severity based on a sole parameter is not recommended [26,65,68,69].

The majority of Doppler methods used for the assessment of left-sided valvular heart disease are applicable when evaluating FTR. However, the TR jet has lower pressure and velocity (the main determinants of the jet momentum) compared to mitral regurgitation [13]. Jet flow and thus color Doppler jet area are governed mainly by the conservation of momentum which is flow (Q) × velocity (V). If Q = effective regurgitant orifice area (EROA) × V, and jet momentum (M) = Q × V, then M = EROA × V^2^. Thus, for the same EROA, the regurgitant volume (RegVol) of a TR jet with a velocity of 2.5 m/s (as frequently recorded in patients without pulmonary hypertension) could be a quarter of the color jet area of a mitral regurgitant jet with a velocity of 5.0 m/s.

Moreover, in patients with A-FTR qualitative signs of TR severity may be misleading: the assumption that the absence of RV dilation usually indicates milder degrees of FTR does not stand true, and the systolic hepatic vein flow reversal could represent the RA dysfunction, and not necessarily FTR severity. Finally, due to the geometrical assumptions regarding single plane VC measurement, and EROA calculation using the PISA method, and since for the same EROA, the RegVol can be quite different with different pressure gradients [40,70], severity quantification in A-FTR is challenging. However, averaged VC width, and VC area by 3DE might overcome the limitations of other semi-quantitative or quantitative parameters that assume the regurgitant orifice is flat and circular, and could be used for A-FTR severity grading when indices provide discordant results [16,70,71].

**Figure 3:**
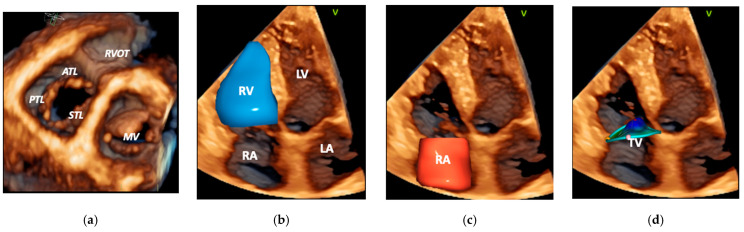
Utility of transthoracic 3DE to assess patients with tricuspid regurgitation. (**a**) Tricuspid valve functional anatomy. (**b**) Right ventricular volume and ejection fraction. (**c**) Right atrial size and function. (**d**) Tricuspid annulus geometry and valve tenting volume and height. Abbreviations: 3DE, three-dimensional echocardiography; ATL, anterior tricuspid leaflet; LA, left atrium; LV, left ventricle; MV, mitral valve; PTL, posterior tricuspid leaflet; RA, right atrium; RV, right ventricle; RVOT, right ventricular outflow tract; STL, septal tricuspid leaflet; TV, tricuspid valve.

Structural parameters (TV morphology, TA size, RV, and RA size) need to be evaluated and 3DE is the most accurate echocardiography technique for this task (Figure 3) [70,72]. 3DE allows to precisely identify the number, morphology, and motion of the different TV leaflets [40,69,73,74,75], which is key to select the optimal devices for transcatheter repair procedures [76].

Additionally, 3DE can easily visualize the structures surrounding the TV, which may serve as landmarks for TV interventions or may have implications for TR, such as the inferior and superior vena cava, the CS inflow, the RV outflow tract, the ascending aorta [32,70].

Due to the complex, 3D configuration and variable spatial orientation of the TA, and since both the 2DE view and the timing of the measurement during the cardiac cycle significantly influence TA size [77,78], 3DE should be the first-line modality for TA sizing in patients with FTR. Since the TA dilates more antero-posteriorly in FTR [79], the greatest TA is unlikely to be identified in the 2D apical 4-chamber view as recommended by the current guidelines [36,40,73]. Furthermore, slight variations in transducer position from apical 4-chamber to RV-focused view results in relatively large differences in TA diameter measurements, and both the absence of anatomical landmarks, as well as the non-circular shape, make 2DE TA linear dimension less reproducible across different studies made in the same patient [36,73]. 3DE provides a precise assessment of the actual TA dimensions (linear, non-planar area, and perimeter), eliminating the geometrical assumptions and absence of anatomical landmarks that characterize 2DE [36,80,81].

**Figure 4:**
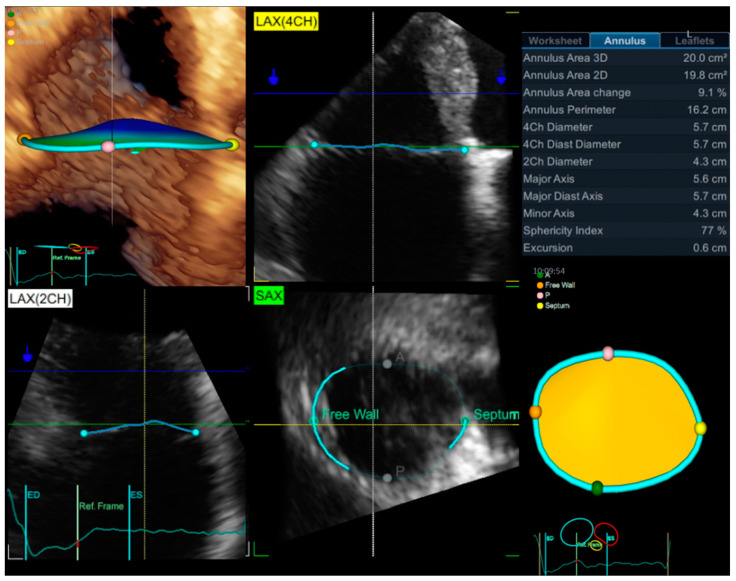
Evaluation of tricuspid annular size and shape by 4D Auto TVQ software ((EchoPac v204, GE, Horten, Norway). Abbreviations: 2Ch, apical 2-chamber view; 2D, two-dimensional echocardiography; 3D, three-dimensional echocardiography; 4Ch, apical 4-chamber view; A, anterior; ED, end-diastole; ES, end-systole; LAX, long axis; P, posterior; SAX, short axis.

A semi-automated 3DE dedicated software for the quantification of TV size and morphology has recently been developed (Figure 4) [36]. The feasibility of TA measurements using this software is high, even in presence of irregular heart rhythms such as AF, and preliminary validation of this software has already been reported [82]. This software package provides various important parameters for TV characterization which are key elements in recognizing the prevalent mechanism of FTR and properly selecting the device used to treat TR [81] (i.e.,: 2D and 3D TA area, TA perimeter, 4- and 2-chamber systolic and diastolic diameters, major and minor axis, sphericity index, longitudinal displacement of the TA during the cardiac cycle, leaflets coaptation point height, tenting volume, maximal tenting height [42]).

While 2DE may also significantly underestimate right heart chambers’ sizes due to foreshortening or geometrical assumptions, 3DE-derived methods allow a more precise and reliable measure of both the RV and the RA [72,83,84].

Lastly, several echocardiographic features have been correlated with procedural failure, suboptimal results, or worse outcomes after transcatheter edge-to-edge TV repair, and they should be assessed when considering patient eligibility. These factors are the presence of more severe leaflets tethering, higher tenting volume, greater coaptation depth (>1 cm), large TR coaptation gap size (>7.2 mm), and non-central/non-anteroseptal TR jet [60,61,85,86]. The number of TV leaflets will also affect TTVIs’ outcomes [87]. Conversely, conventional echocardiographic parameters used to assess RV function and systolic pulmonary artery pressure may not predict clinical outcomes after transcatheter valve repair [88].

### 3.2. Cardiac Magnetic Resonance, Cardiac Computed Tomography, and Fusion Imaging

The reference imaging technique for evaluating RV size and systolic function, which are important elements in distinguishing between A-FTR and V-FTR, is CMR. Moreover, CMR can provide accurate TR severity grading based on the measurement of the RegVol and the regurgitant fraction, indirectly calculated by subtracting the pulmonic forward volume from the RV stroke volume [68], or directly measured by the use of either standard phase-contrast sequences [89,90], or by innovative 4D-flow velocity-encoded approaches, using whole-heart free-breathing sequences [91,92]. However, CMR is not well suited for assessing the TV leaflets due to their thinness. Conversely, CCT, that is characterized by high spatial resolution, is not only the ideal method to assess TA dimensions and the anatomic considerations that are fundamental when planning transcatheter procedures targeting the TA [2,3], but also allows visualization of TV leaflets, precise assessment of RV dimensions and sub-valvular structures—trabeculations, papillary muscles, moderator band, and direct measurement of EROA by multiplanar reformations analysis [32,93,94].

CMR and CCT acquisitions and postprocessing can be hampered by motion artifacts in AF patients. However, the use of new pulse sequences and imaging reconstruction algorithms allow real-time free-breathing cine sequences with good spatial and temporal resolution, and can provide precise results in the CMR evaluation of the right heart chambers [95,96,97]. Similarly, CCT data acquisition impacts the quality of the images, and it is of paramount significance. An optimal contrast enhancement of the right heart using a dedicated CCT contrast protocol [98] allows the acquisition of images of good quality even in the challenging scenarios of AF. Moreover, the dedicated CCT protocols used to study the TV [99] limit the use of contrast media, avoid artifacts, and provide a homogeneous opacification of the right heart. Accordingly, CCT has emerged as a standard imaging modality that provides incremental value in establishing patient eligibility and proper device sizing in the setting of TTVI [64], and it represents an ideal alternative to CMR for the measurement of right heart dimensions in patients with noncompatible intracardiac devices or contraindications to CMR [83,100].

Finally, although CCT is the method of choice for assessing TA dimensions [32,37,101], reevaluating TA dimensions and TR severity by TEE at the start of the procedure is of paramount importance since TR severity and TA size are dynamic and load-dependent. TEE is also used to identify anatomic markers that are not visible at fluoroscopy, such as the CS or the venae cavae [22,32]. Furthermore, fluoroscopy is required to position wires and guiding catheters during TTVIs. However, fluoroscopy does not allow the visualization of the TV or landmark structures. Therefore, fusion imaging (superimposing echocardiographic or CCT images on fluoroscopic projections) represents a novel alternative for imaging patients undergoing TTVIs [102,103,104,105].

## 4. Conclusions

A-FTR is a distinct pathophysiological and clinical entity, with different needs of clinical management as well as choice of TV intervention, and most likely with different outcomes than V-FTR. Defining the etiology of FTR, and distinguishing between A-FTR and V-FTR plays a crucial role in the management and selection of the patients for TTVIs. Multimodality imaging is key for confirming the indication for the interventions, the guiding and monitoring of TTVIs, and in the assessment and follow-up of the results of the procedure.

## Data Availability

No new data were created or analyzed in this study. Data sharing is not applicable to this article.
